# Pathogenesis diagnosis of a pediatric patient suffering from multi-organ abscesses

**DOI:** 10.1186/s13000-023-01360-6

**Published:** 2023-07-29

**Authors:** Mingkun Wu, Haijuan Xiao, Yan Xiao, Tianming Chen, Xinming Wang, Xia Xiao, Ying Wang, Jianwei Wang, Lili Ren, Gang Liu

**Affiliations:** 1 National Health Commission of the People’s Republic of China Key Laboratory of Systems Biology of Pathogens and Christophe Mérieux Laboratory, Institute of Pathogen Biology, Chinese Academy of Medical Sciences (CAMS) & Peking Union Medical College, No.9 Dong Dan San Tiao, Dongcheng District, 100730 Beijing, P. R. China; 2 Department of Infectious Diseases, Key Laboratory of Major Diseases in Children, Beijing Children’s Hospital, Ministry of Education, Capital Medical University, National Center for Children’s Health, No.56 NanLiShi Road, Xicheng District, 100045 Beijing, P. R. China; 3 Key Laboratory of Respiratory Disease Pathogenomics, Chinese Academy of Medical Sciences and Peking Union Medical College, 100730 Beijing, P. R. China

**Keywords:** Multi-organ abscesses, *Bacteroides fragilis*, Anaerobic bacteremia, Human adenovirus 2 (HAdV-2), Metagenomics next-generation sequencing (mNGS)

## Abstract

A pediatric patient suffered from renal and hepatic abscesses (through hepatorenal space) and recovered by antibiotics and other therapies. By clinical analysis, the multi-organ abscesses might be caused by bloodstream-disseminated infection. In order to identify the pathogen, we collected kidney biopsy tissue, swabs, and plasma samples, and used metagenomics next-generation sequencing (mNGS) and some traditional methods. The results revealed that polymicrobial especially anaerobic bacteremia *(Bacteroides fragilis*, et al.) contributed to the abscess formation. What is more, systematic human adenovirus C (HAdV-C) infection was shown, and the virus was isolated. The titer of HAdV-2 neutralizing antibodies was 1/4 in the plasma after symptoms onset. Although the exact mechanism of HAdV-2 infection in multiple abscess formation has not been clarified, the case of multi-organ abscesses in the context of polymicrobial especially anaerobic bacteremia and HAdV infection in healthy children is infrequent.

**Supplementary Information:**

The online version contains supplementary material available at 10.1186/s13000-023-01360-6.

## Introduction

Multi-organ abscesses could affect multiple physiological functions and cause adverse clinical outcomes [1]. However, pathogen isolation is usually a challenge. Some diagnostic approaches, such as serology, molecular test, and metagenomics next-generation sequencing (mNGS) methods, have been developed as powerful tools for pathogen surveillance and identification.

## Case presentation

**Fig. 1:**
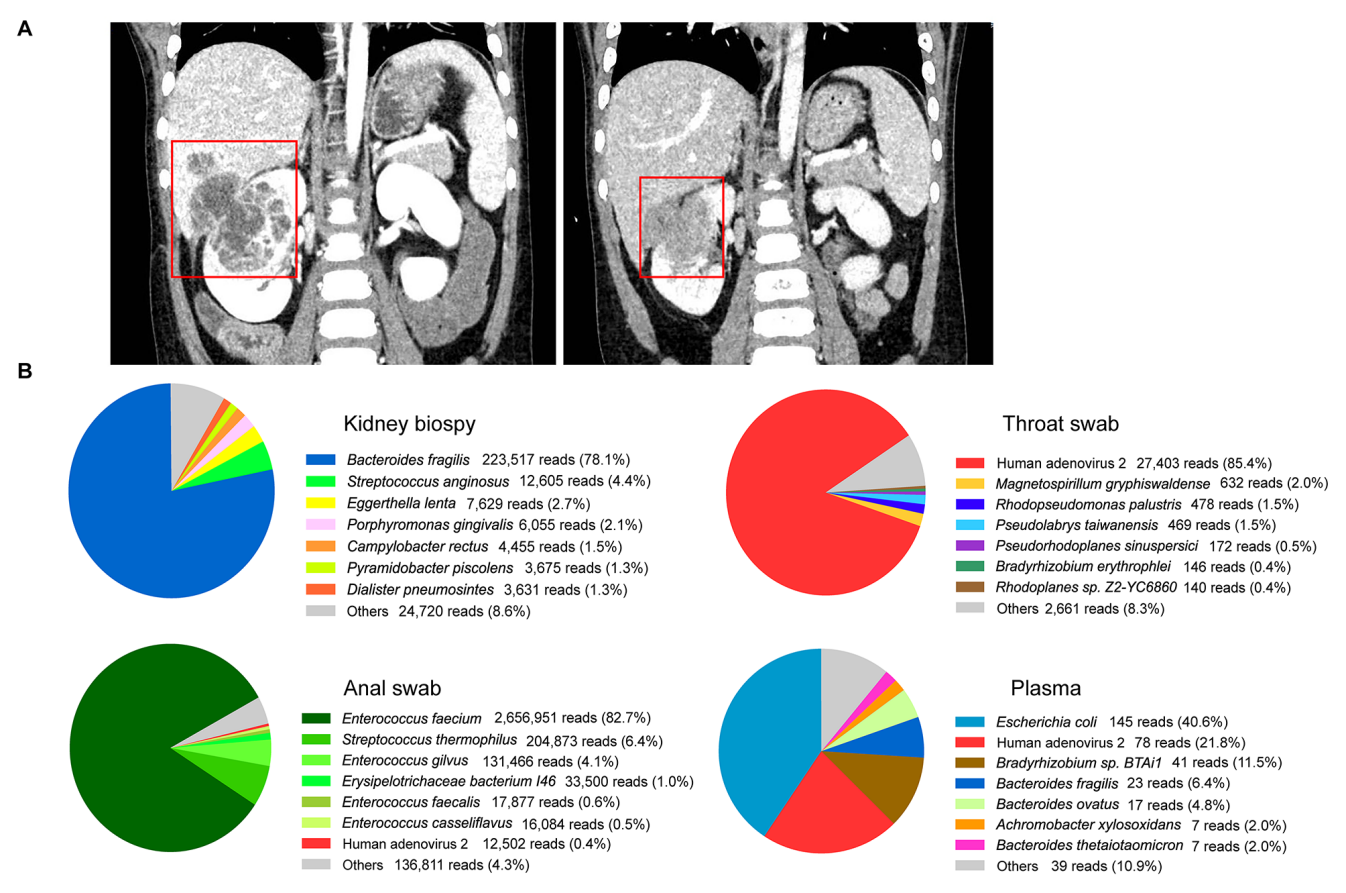
The imaging manifestations and pathogen detection of the pediatric patient suffering from multi-organ abscesses. **(A)** The space-occupying lesion of the right kidney with a patch-shaped low-density area inside extends to the liver (with a mixed low-density area inside) through hepatorenal space before treatment (left) and the lesions improved after two months’ treatment (right) by contrast-enhanced computed tomography (CT). **(B)** The profiles of the microbial species (top 7) in the kidney biopsy, throat swab, anal swab, and plasma by metagenomics next-generation sequencing (mNGS).

In our study, a 2.5-year-old boy was admitted to hospital suffering from recurrent fever for 16 days, with transient abdominal pain, vomit, and diarrhea in the course of disease. He received only supporting treatment. This boy had no known immunosuppression status and denied a history of recurrent infections, trauma or animal exposure. On admission, routine blood examination showed leukocytosis (white blood cell (WBC), 11.96 × 10^9^/L), elevated C-reactive protein (CRP, 120 mg/ml), and increased erythrocyte sedimentation rate (ESR, 140 mm/h). The contrast-enhanced computed tomography (CT) showed a space-occupying lesion of the right kidney with a patch-shaped low-density area inside, which extended to the liver (with a mixed low-density area inside). The enhancement pattern of hepatic lesions was similar to the renal lesions: the wall enhancement became evident gradually, with no enhancement of the low-density focus inside, suggesting multi-organ abscesses (Fig. 1A). The abdominal ultrasound revealed multi-masses in the right posterior liver (2.3 × 2.1 × 1.8 cm), hepatorenal space (4.3 × 4.0 × 3.3 cm), and right kidney (6.3 × 5.7 × 4.8 cm), with varieties of small patch shaped turbid liquid areas inside. According to clinical analysis, the renal abscess occurred first, and then the liver abscess occurred through hepatorenal space. The case had no gastrointestinal tract, oral cavity, brain, or lung lesions. No urinary tract or biliary tract anomalies were found. The urine tests were all normal, and no intraperitoneal infection was found, indicating that multi-organ abscesses might be caused by bloodstream-disseminated infection.

This patient was treated with meropenem for 35 days, combined with metronidazole (added after the pathogen result) for 1 month, and then cefoperazone sulbactam monotherapy for another 21 days after hospitalization. Body temperature became normal after 15 days of treatment. The WBC and CRP returned to normal, and ESR declined to 25 mm/h. The ultrasound showed apparent shrinking of the lesions in the liver (1.1 × 0.8 × 0.9 cm), hepatorenal space (2.3 × 1.4 × 2.3 cm), and right kidney (3.4 × 2.3 × 2.5 cm). The patient received oral amoxicillin and clavulanate potassium after being discharged from hospital.

To identify the potential pathogens, we performed an ultrasound-guided kidney biopsy, while a throat swab, anal swab, and plasma were collected simultaneously. The deep sequencing analysis suggested coinfection of several bacteria in the kidney tissue, including anaerobic species (*Bacteroides fragilis*, 223,517 reads; *Eggerthella lenta*, 7,629 reads; *Porphyromonas gingivalis*, 6,055 reads; et al.), Enterobacteriaceae (*Escherichia coli*, 317 reads) and facultative anaerobic streptococci (*Streptococci anginosus*, 12,605 reads) (Fig. 1B). *Bacteroides fragilis* and *Escherichia coli* were also detected from plasma by mNGS. Combined with clinical analysis and the sequencing analysis of plasma, polymicrobial especially anaerobic bacteremia could contribute to the abscess formation. However, blood culture for anaerobic bacteria was negative, which might be associated with the use of antibiotics. Notably, human adenovirus C (HAdV-C) reads were also detected from the throat swab, anal swab, and plasma using DNA deep sequencing (Fig. 1B). Moreover, the replication of HAdV-C was found in the kidney tissue (7 RNA reads), respiratory tract (11,294 RNA reads) and gastrointestinal tract (36 RNA reads) using metatranscriptomics sequencing. These results suggested systematic HAdV-C infection. The assembled genome length of this strain (HAdV-2/Beijing/2022/MA-TS) was 35,714 bp, which was related to the HAdV-2 in phylogenetic analysis (Supplementary Fig. 1). The HAdV-2 was isolated from the throat swab and anal swab using HeLa cells (Supplementary Fig. 2), and the cultured viral genome sequence was consistent with that of the swabs (Supplementary Fig. 1). The titers of HAdV-2 neutralizing antibodies (NAbs) in the plasma collected in 23 days and 25 days post symptoms onset were 1/4 using isolated HAdV-2.

## Discussion

This patient suffered from renal and hepatic abscesses caused by polymicrobial especially anaerobic bloodstream-disseminated infection. Moreover, systematic HAdV-C infection was shown, and the virus was isolated. By using multiple techniques including mNGS, pathogen culture, and serological test, our study showed an unusual case of renal and hepatic abscesses in the context of polymicrobial especially anaerobic bacteremia and HAdV infection in healthy children.

Combining the pathogen results and treatment effectivities, we found the clinical symptoms were improved after the treatment with meropenem and metronidazole, antibiotics for anaerobes. Such findings supported that the polymicrobes especially anaerobes were the causative pathogens for multi-organ abscesses. Based on the clinical, radiological, and ultrasonic analysis, renal abscess was the major focus of infection, and the lesions extended to the liver through hepatorenal space. Our patient had no intraperitoneal infection and no urinary tract infection, therefore, polymicrobial especially anaerobic bloodstream-disseminated infection was considered. The mNGS result of plasma verified bloodstream infection. In fact, there were literature reports for anaerobic bacteraemia (*Bacteroides fragilis* was the predominant anaerobic pathogen, followed by *Clostridium* species and *Propionibacterium* species), liver and kidney abscesses caused by anaerobe (*Clostridium clostridioforme*), and renal abscess caused by anaerobe (*Peptostreptococcus asaccharolyticus*) [2–4]. This patient had no special past history, and did not suffer from oral and gastrointestinal infections. The exact reason for polymicrobial especially anaerobic bloodstream infection was not clarified.

In our patient, HAdV-C was detected from the kidney biopsy tissue, throat swab, anal swab, and plasma using the sequencing method, suggesting systematic HAdV-C infection. HAdV has been found to be colonized in tonsillar lymphocytes in nearly 80% of children, causing asymptomatic to severe infections, such as hepatitis, pneumonia, and encephalitis, especially in immunocompromised patients and transplant recipients [5–7]. Latent HAdV might exist in renal parenchyma in lymphoid tissue and other tissues for years [8]. Although the infection of HAdV-2 was confirmed by viral isolation in our patient, no specific humoral response was established as the titer of NAbs against HAdV-2 was low. Therefore, HAdV-C may not be the pathogen responsible for abscesses.

As for whether HAdV infection was related to multi-organ abscess formation, there was no direct and exact evidence. In a mouse model, HAdV-C infection induced high sensitivity of lipopolysaccharide and staphylococcal enterotoxin-B [6, 9, 10]. Meanwhile, HAdV infection may induce excessive IFN-γ production and IFN-γ-mediated apoptosis, thereby influencing the host’s immune responses [11]. Therefore, HAdV infection might impact the host microbial homeostasis, and lead to opportunistic infections. Moreover, a case of liver abscess with HAdV infection has been reported in a pediatric liver transplant recipient: the necrotic liver tissue surrounding pus showed positive immunohistochemical staining for adenovirus, while the liver tissue of portal inflammation with granulomata showed negative adenovirus immunohistochemical staining [12]. Serum and urine adenovirus PCR were both positive [12]. These mechanisms might contribute to the pathogenesis of HAdV-2 infection in multi-organ abscesses: HAdV infection might be a precipitating factor for abscess formation. Anyway, the case with multi-organ abscesses in the context of polymicrobes especially anaerobes and HAdV infection in healthy children is infrequent. However, the clinical role of HAdV-2 in abscess formation still needs to be further clarified.

In this study, we identified infectious agents causing multi-organ abscesses in a pediatric patient with unknown etiology by combining the mNGS technique, traditional pathogen culture, and serological analysis. Pathogen coinfection in the abscess was confirmed. Our findings emphasize the combination of mNGS and other traditional methods in the pathogen diagnosis process to help clarify the causative agents.

## Electronic supplementary material

Below is the link to the electronic supplementary material.

Supplementary Material 1
