# Supplementary material for: Pathogenesis diagnosis of a pediatric patient suffering from multi-organ abscesses
Source: Diagn Pathol. 2023 Jul 29;18:85. doi: 10.1186/s13000-023-01360-6 (PMC10386630; doi:10.1186/s13000-023-01360-6)

**Pathogenesis diagnosis of a pediatric** **patient suffering from multi-organ abscesses**

Mingkun Wu^1,*^, Haijuan Xiao^2,*^, Yan Xiao^1,3^, Tianming Chen^2^, Xinming Wang^1^, Xia Xiao^1^, Ying Wang^1^, Jianwei Wang^1,3,†^, Lili Ren^1,3,†^, Gang Liu^2,†^

* Joint first authors and contributed equally

† Joint corresponding authors and contributed equally

^1^ NHC Key Laboratory of Systems Biology of Pathogens and Christophe Mérieux Laboratory, Institute of Pathogen Biology, Chinese Academy of Medical Sciences (CAMS) & Peking Union Medical College, Beijing, China.

^2^ Department of Infectious Diseases, Key Laboratory of Major Diseases in Children, Ministry of Education, Beijing Children's Hospital, Capital Medical University, National Center for Children's Health, Beijing, China.

^3^ Key Laboratory of Respiratory Disease Pathogenomics, Chinese Academy of Medical Sciences and Peking Union Medical College, Beijing, 100730, P. R. China.

**Supplementary materials**

**Methods**

**Patient and samples**

The study was approved by the Ethics Committee of Beijing Children’s Hospital, Capital Medical University, and performed according to the Declaration of Helsinki. Written informed consents were obtained from the parents. Clinical data and samples (including kidney tissue, throat swab, anal swab, and plasma) were collected from the patient with multi-organ abscesses.

**Library construction for deep sequencings**

The microbial nucleic acids in kidney biopsy, throat swab, anal swab, and plasma were extracted using NucliSENS miniMAG (bioMérieux, France). The nucleic acids concentration was measured using Qubit Fluorometer (Invitrogen, U.S.). The DNA libraries for metagenomics sequencing were constructed using TruePrep Flexible DNA Library Prep Kit for MGI (Vazyme, China). The RNA libraries for metatranscriptomics sequencing were constructed using Trio RNA-seq kit (NuGEN Technologies, U.S.).

**Virus isolation and culture**

Human adenovirus 2 (HAdV-2) was isolated from the throat swab and anal swab by inoculation onto HeLa cells (ATCC, #CCL-2). For validation, the nucleic acids in the virus fluid were extracted using NucliSENS miniMAG and DNA libraries were constructed. The virus was propagated for use in this study.

To determine the virus titer, gradient dilution (1/10-1/10^8^) was performed using Dulbecco's modified Eagle medium (DMEM; Gibco, U.S.). 100 μL virus diluent was incubated with 10^4^ HeLa cells in 96-well plates for 1 h at 37°C and 5% CO_2_ concentration. Non-infected cells were used as negative controls, and eight duplicate wells were used for each dilution. The unbound virus was washed away after 1 h, and cells were then cultured with fresh medium supplemented with 2% fetal bovine serum (FBS; HyClone, U.S.). The cytopathic effect (CPE) images were obtained 5 days after incubation and analyzed using a laser scanning confocal microscope (Zeiss LSM 800, Germany). The 50% tissue culture infective dose (TCID_50_) was calculated using the Reed-Muench method.

**Neutralization assay**

The presence of neutralizing antibodies (NAbs) in the plasma samples was assessed using microneutralization assays. The plasma samples were inactivated for 30 minutes at 56°C. A serial two-fold dilution of plasma (1/4-1/128) was preincubated with HAdV-2 at 100 TCID_50_ for 1 h at 37°C. The virus-plasma mixture was then added to HeLa cells and incubated for 1 h at 37°C and 5% CO_2_ concentration. The unbound virus was washed away after 1 h, and cells were then cultured with fresh medium supplemented with 2% FBS. Four duplicate wells were used for each plasma dilution, and viral back-titration was done. CPE was assessed 5 days after incubation and NAbs titer was calculated using the Reed-Muench method.

**Statistical analysis**

The low-quality sequences were filtered using fastp v0.20.1. The human reads were first depleted using Kneaddata v0.10.0, and the remaining human reads were excluded in subsequent analyses. The microbial reads were mapped using Kraken2 v2.1.2. The whole HAdV-2 genome was obtained using SPAdes genome assembler v3.15.3 and Prokka v1.12 was used for genome annotation. The penton gene, fiber gene, and whole genome were aligned with other HAdV genotypes in Mafft v7.310 separately, and phylogenetic trees were constructed by maximum-likelihood using Iqtree2 v2.2.0.

**Supplementary figures**

**Fig. S1 Phylogenetic analysis of different HAdV strains based on genome, penton gene, and fiber gene.**

**(A)** The phylogenetic tree of viral genome obtained from the throat swab (HAdV-2/Beijing/2022/MA-TS), and penton gene **(B)** and fiber gene **(C)** from the anal swab isolate (HAdV-2/Beijing/2022/MA-AS isolate) using the maximum likelihood method. A, B, C, D, F, and G represent genotypes.

**
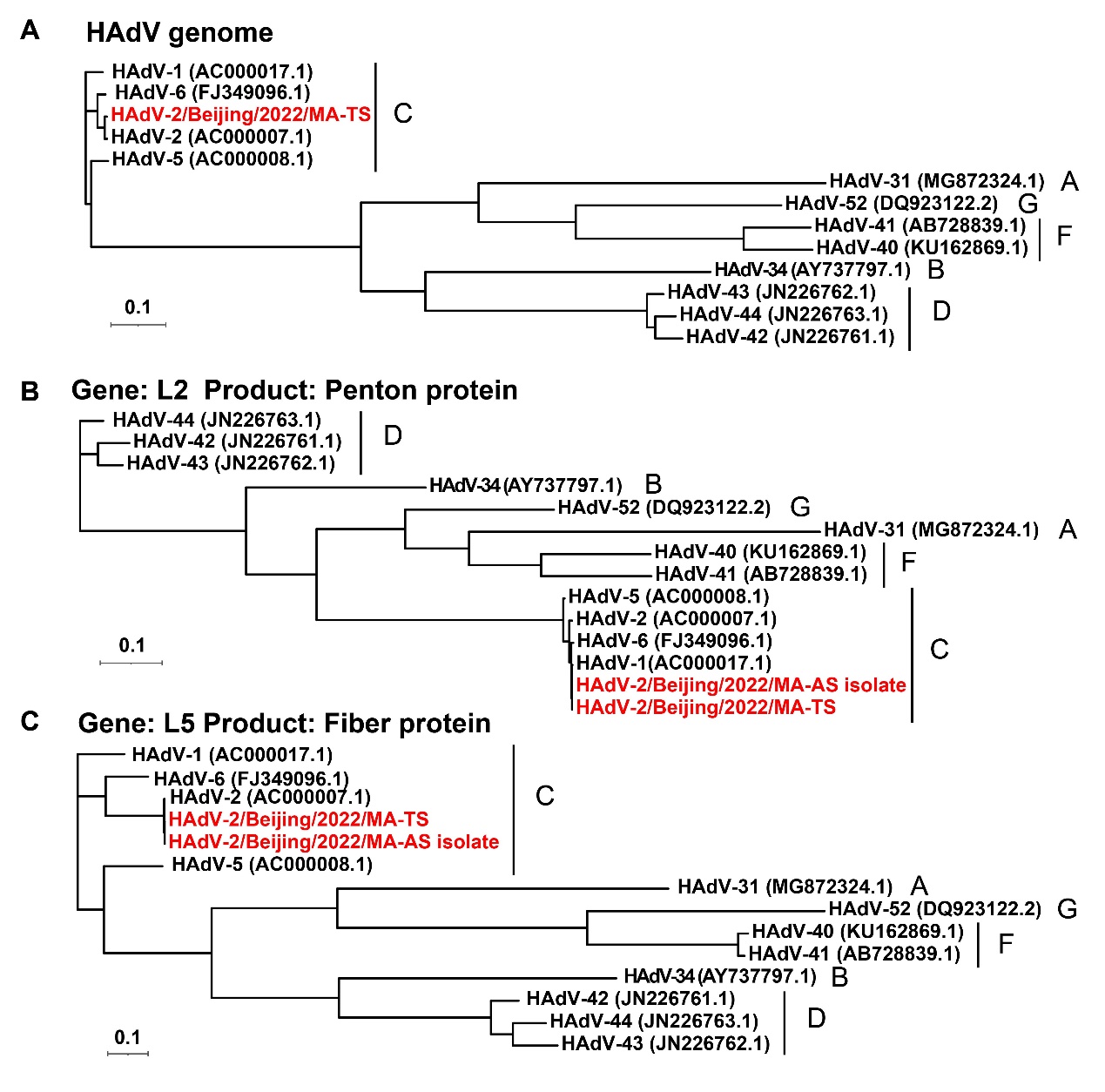
**

**Fig. S2 The isolation of human adenovirus 2 from the throat swab and anal swab of the pediatric patient with multi-organ abscess**.

**(A)** The cytopathic effect (CPE) caused by isolated virus on the HeLa cells **(B)** The cell control.


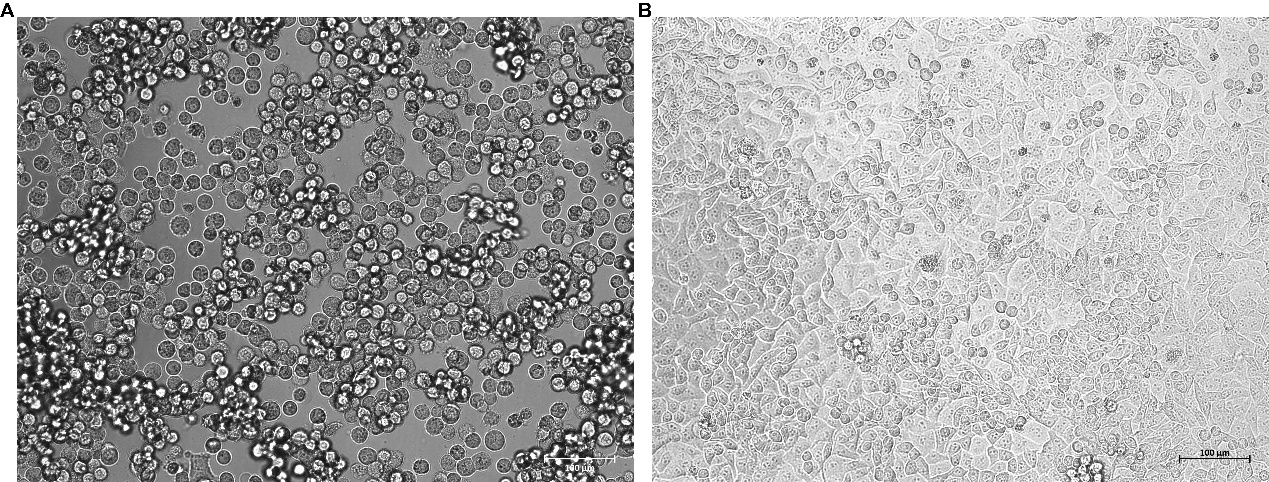

Supplement: Supplementary file 1 — Supplementary Material 1 [file 13000_2023_1360_MOESM1_ESM.docx]
